# Validation and selection of ODE based systems biology models: how to arrive at more reliable decisions

**DOI:** 10.1186/s12918-015-0180-0

**Published:** 2015-07-08

**Authors:** Dicle Hasdemir, Huub C.J Hoefsloot, Age K. Smilde

**Affiliations:** Biosystems Data Analysis Group, Swammerdam Institute for Life Sciences, University of Amsterdam, Amsterdam, The Netherlands; Netherlands Metabolomics Centre, Leiden, The Netherlands

**Keywords:** Kinetic models, ODE, Differential equations, Model validation, Model selection, Cross validation, Hold-out validation

## Abstract

**Background:**

Most ordinary differential equation (ODE) based modeling studies in systems biology involve a hold-out validation step for model validation. In this framework a pre-determined part of the data is used as validation data and, therefore it is not used for estimating the parameters of the model. The model is assumed to be validated if the model predictions on the validation dataset show good agreement with the data. Model selection between alternative model structures can also be performed in the same setting, based on the predictive power of the model structures on the validation dataset. However, drawbacks associated with this approach are usually under-estimated.

**Results:**

We have carried out simulations by using a recently published High Osmolarity Glycerol (HOG) pathway from *S.cerevisiae* to demonstrate these drawbacks. We have shown that it is very important how the data is partitioned and which part of the data is used for validation purposes. The hold-out validation strategy leads to biased conclusions, since it can lead to different validation and selection decisions when different partitioning schemes are used. Furthermore, finding sensible partitioning schemes that would lead to reliable decisions are heavily dependent on the biology and unknown model parameters which turns the problem into a paradox. This brings the need for alternative validation approaches that offer flexible partitioning of the data. For this purpose, we have introduced a stratified random cross-validation (SRCV) approach that successfully overcomes these limitations.

**Conclusions:**

SRCV leads to more stable decisions for both validation and selection which are not biased by underlying biological phenomena. Furthermore, it is less dependent on the specific noise realization in the data. Therefore, it proves to be a promising alternative to the standard hold-out validation strategy.

**Electronic supplementary material:**

The online version of this article (doi:10.1186/s12918-015-0180-0) contains supplementary material, which is available to authorized users.

## Background

Ordinary differential equation (ODE) based kinetic models are able to capture all of the available kinetic information regarding a biological system. Therefore, they are used extensively in systems biology especially for the purpose of predicting time dependent profiles and steady state levels of biochemical species in conditions where experimental data is not available. Examples from the literature show that there is a common path taken by the modeling community for the construction and the analysis of ODE based systems biology models. The first step is to define the model structure and the associated kinetics. Due to serious concerns about the validity of model structures and kinetics, many studies include the parallel development and analysis of multiple alternative model structures [1–3]. The second step is the estimation of the unknown model parameters by fitting the model to the data using global and local minimization algorithms. Data here are usually *in vivo* time series concentration data of the observable biochemical species included in the model. At this step, uncertainty in the estimated values of the model parameters can also be quantified by constructing confidence intervals [4–6]. Last but not least, models are assessed for the quality of their fit to the data and for their predictive power on independent data. Independent data are datasets that were not used for parameter estimation. Selection between alternative model structures can also be performed at this step. A complete modeling cycle includes all these steps to achieve sufficiently good models [7, 8].

A good model has to be sufficient both in explaining the data on which it was built and in predicting independent data [9]. The first is taken into account mostly by likelihood ratio tests which can be used to reject models based on the quality of fit to the data [10–13]. The second aspect has been considered in conceptually two different ways. The first approach uses a penalized likelihood based metric such as Akaike’s (AIC) [14] or Bayesian Information Criterion (BIC) [15]. This metric is calculated using the whole dataset for parameter estimation but provides an expected value of the prediction error on an independent dataset. Therefore, it makes selecting the true complexity of a model possible because unnecessarily complex models are poor in predicting independent datasets. However, it is an ‘in-sample’ measure which means that the expected prediction error is valid only for the exact same experimental conditions as of the parameter estimation dataset [10]. Predicting the kinetics of the biological system under different experimental conditions is the very purpose of kinetic models, though. Therefore, modelers would like to show that the newly built model is good in qualitative or quantitative prediction of experimental data that was collected at different experimental conditions. This strategy which uses data at different experimental conditions as validation data constitutes the second approach to assess the prediction error [8, 16].

Different experimental conditions are usually based on the following scenarios:

- Inhibition of enzymes.
- Reduction of protein levels by RNAi mediated suppression.
- Gene deletions.
- Over-expression of genes in gene networks.
- Dose-response experiments in which different doses of triggering chemicals are used to stimulate the system.

These validation scenarios are popularly applied since the common goal of the modelers is to demonstrate the models’ competency under challenging conditions. Estimating the parameters of a model in certain experimental conditions and showing their competency in other conditions within these scenarios requires multiple datasets under different conditions and, therefore, it is an example to the hold-out validation strategy. That is, a pre-determined set of conditions are held out of the training data and used as validation data instead. However, rules about the application of this strategy are not straightforward.

There are potential pitfalls associated with the application of hold-out validation strategies in the validation and selection of kinetic systems biology models. These arise due to the lack of a satisfying answer to the question: *which part of the dataset should be held out of the parameter estimation and instead should be used as the validation dataset?* We carried out simulations to demonstrate the phenomena that hinder us from giving satisfactory answers to this question which can also be referred to as the problem of selecting an appropriate hold-out partitioning scheme for the data. The problem arises due to incomplete biological knowledge of the system and unknown true values of the model parameters. This makes the problem a paradoxical one since our knowledge about the system will never be complete and the true values of the model parameters are themselves what we are looking for. However, statistics literature offers an established method which is independent of this knowledge, namely cross-validation.

Cross-validation (CV) is a resampling method traditionally used for model selection, determining the optimal complexity of a model or assessment of its generalizability in statistics [17, 18]. It is based on the partitioning of the data in training and test sets. The training set is used to build the model and the predictions of the model on the test set are used for model assessment. Since the test set is completely independent of the parameter estimation process, selection will not be biased towards more complicated models. The efficiency of cross-validation and its difference from hold-out validation strategy lies in the fact that the partitioning is made not in a pre-determined but in a random way and the procedure is repeated multiple times so that each partition can be used as test set at least once. Different variants of CV exist such as leave-one-out, k-fold and stratified k-fold cross-validation. A comprehensive evaluation and comparison of these methods can be found in [19–21] for classifier selection and in [22] for the selection of regression models.

CV has been applied in different ways in the ODE based modeling framework. Partitions can consist of different experiments (such as different cell types, experimental conditions or cultures), data belonging to different biochemical species in the same experiment or different data points within the time profile of the same biochemical species. In [23], the authors present an example for the latter. In this work, prediction errors on test sets obtained by using an ODE based model are compared to the residuals from an unsupervised data analysis method which does not make any use of biochemical knowledge. Better predictions found by using the unsupervised principal components analysis (PCA) method give hints on the low informative level of the ODE model leading to a rejection of the proposed ODE model structure. CV by using different species from the same or different cultures with different experimental conditions was considered by [24]. In that study, prediction errors were used to select not between two single models but between two families of models each constituted of models with slightly different topologies. Both approaches use a k-fold stratified partitioning scheme in which time points or species were approximately equally distributed between k different partitions. The prediction errors from different test sets are averaged for the final measure of the predictive power.

Existence of only very few examples like we mentioned above show that CV has been highly neglected in the field. Also, the risks associated with the hold-out validation strategy have been underestimated. The conceptual differences between the two methods and the difference between their outcomes have not been presented in detail. Therefore, with this paper we aim to present a detailed comparison of the hold-out and cross-validation methods by using simulations and emphasize the advantages of CV over hold-out partitioning schemes. More details on our implementation of CV are given in the Methods section.

The reason for choosing simulations for our demonstrative purposes is that simulations and synthetic data allows us to know the ground truth, in this case the true model parameters and the true model structure. Therefore, we can analyze the results we achieved in different partitioning schemes in a comparative manner. We mainly look at the effect of different partitioning schemes in the outcome of model validation and selection. However, we report also results related to its effect on parameter estimation which is very influential on validation and selection in order to present a complete explanation.

## Methods

### Simulated data

**Fig. 1:**
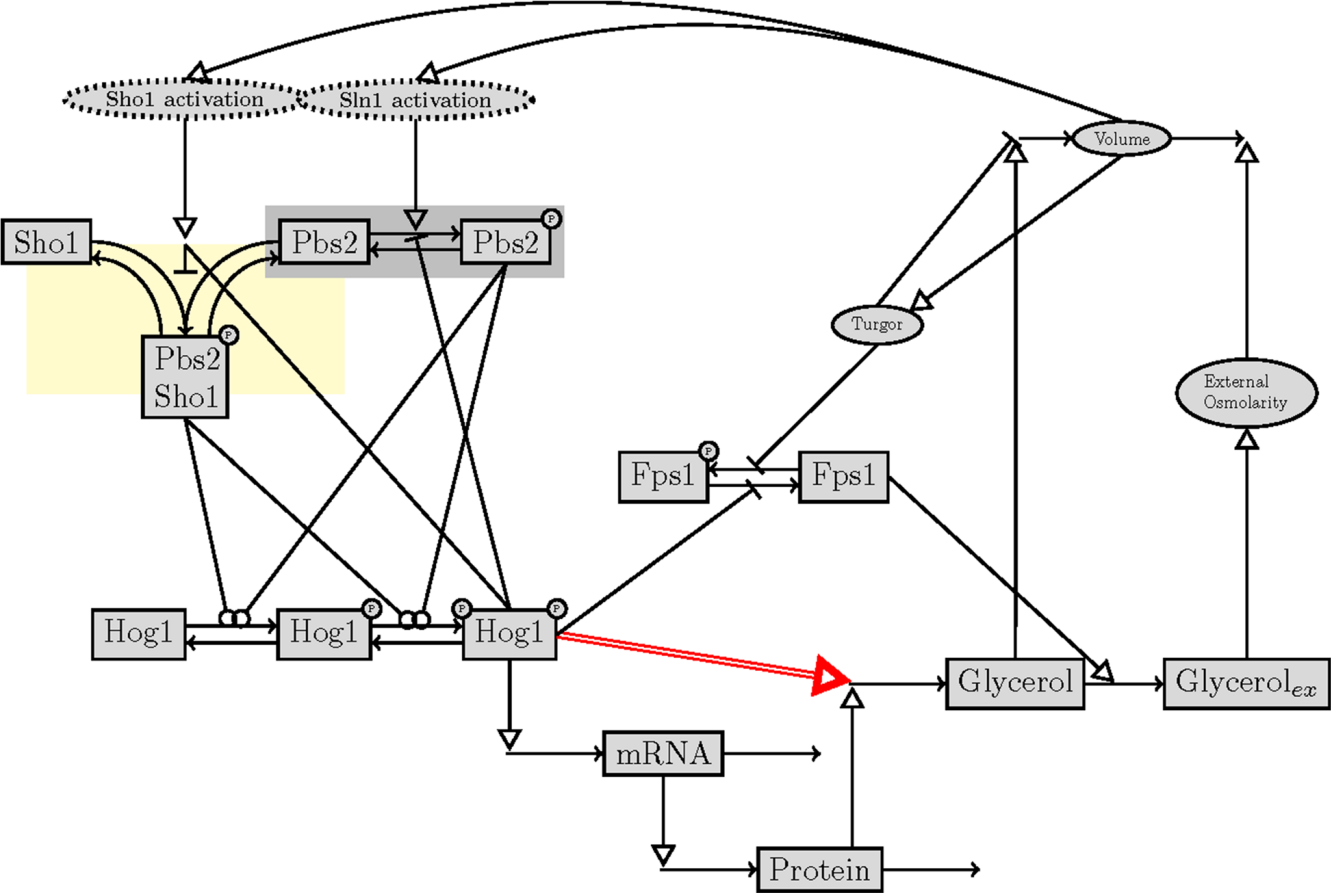
The pathway topology proposed in [25]. We used this model as our true model and generated data based upon it. The black lines with small arrow tips depict the transition between different species in the model like production, degradation or complex formation. The black lines with open circle tips depict the phosphorylation process by kinases. The lines with open triangle tips show activating regulatory interactions whereas lines with blunt ends show deactivating regulatory interactions. The red colored double arrow denotes the post translational regulation of glycerol production by the active phosphorylated Hog1 protein. We did not consider this regulatory interaction in our simplified model. The dotted ellipses in the upper left hand corner indicate the two different upstream activation routes important in our study. Parts of the pathway whose parameters were affected by the choice of the partitioning scheme were highlighted yellow and gray. We explained the changes in the parameters of those regions in our results section. (Figure adopted from [23])

We used the high osmolarity glycerol pathway model in *S.cerevisiae* which was presented as the best approximating model in [25] (see Fig. 1) to generate synthetic data. The model is available in Biomodels Database [26] with the accession number MODEL1209110001. The readers are referred to the original paper for the details of the model structure.

The pathway can be triggered by using an NaCl shock and is activated via two parallel upstream signaling routes. The activity of the upstream routes is encoded by a binary input parameter which indicates that the route is either active or not. The level of the NaCl shock is also an input parameter which can be manipulated. Therefore, the model can be used also for deletion mutants where only one of the signaling routes is active, following different doses of NaCl shock, by changing only those two input parameters. It includes additional 20 free parameters which can be estimated from data.

**Fig. 2:**
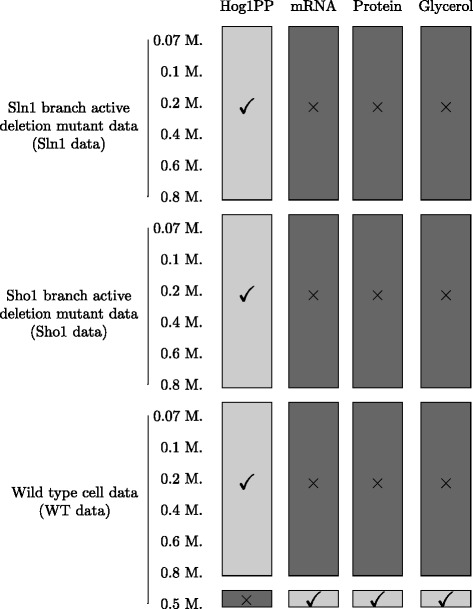
Experimental conditions under which the data was generated. Check marks indicate the measurements that were performed. Each row shows a different dose in a different cell type whereas columns are for different biochemical species measured. Hog1PP data consists of 18 subsets (6 different doses and 3 different cell types) and is the main subject of variability between different partitioning schemes that we evaluated

We mimicked the real experimental conditions used in [25] when generating the data. These include different cell types and different NaCl doses. The different cell types were deletion mutants in which only the signaling branch through Sln1 activation or Sho1 activation was active and the wild type cell in which both branches were active (Fig. 1). The different NaCl shock levels ranged between 0.07 and 0.8 M (Fig. 2). The data consisted mainly of the ratio of the active phosphorylated Hog1 protein to the maximum Hog1 protein level observed in the wild type cell which was expressed as a percentage. The Hog1 protein phosphorylation percentage data (Hog1PP data) from 3 cell types and 6 doses formed 18 different subsets of Hog1PP data. We used different subsets for parameter estimation and model validation/selection each time within different data partitioning schemes which we explain in detail in the following section. Concentration data of other species in the model were essential for the estimation of the parameters downstream from the Hog1 protein. For this reason, measurements of mRNA, protein and glycerol levels at 0.5 M. NaCl shock were always a part of the training dataset. Therefore, the terms ’validation data’ and ’training data’ refer only to Hog1PP data, throughout the text.

We generated 100 different realizations of synthetic data by adding error to the time profiles obtained by the model. We added heterogeneous noise where the noise term for each concentration value was drawn from a normal distribution with a standard deviation equal to 10 *%* of the concentration value itself which reflects realistic noise levels and structure for these type of experiments. The time series data contained 15 time points during a course of 160 minutes.

### Data partitioning schemes

#### Hold-out partitioning schemes

**Fig. 3:**
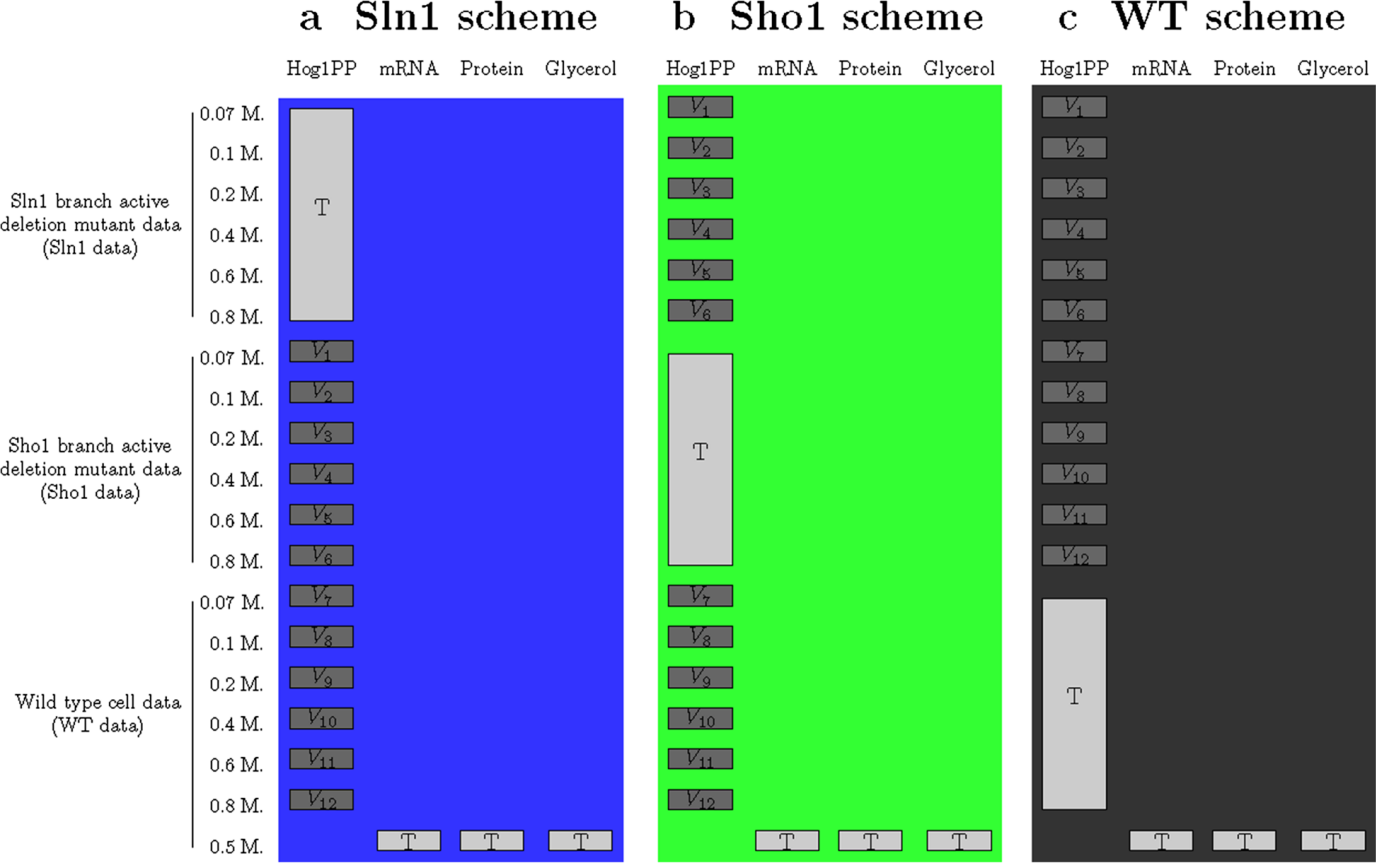
Scenario 1 partitioning schemes. Light gray colored boxes show parts of the data which we used as the training set (T) for parameter estimation. Dark gray colored boxes show parts which we used as validation sets (V). Different background colors represent different partitioning schemes and are consistent with the colors used in the graphs in the Results and discussion section. Each partitioning scheme offered the use of six subsets of the Hog1PP data as the training set and the remaining twelve subsets of the Hog1PP data could be used for validation, separately. The training set included either (**a**) Sln1 data, (**b**) Sho1 data or (**c**) WT data

In this work, we evaluate the performance of the hold-out validation partitioning schemes based on two most popularly applied challenge scenarios: gene deletions and dose-response experiments. The first scenario in our study mimics a gene deletion challenge. In each scheme of this scenario (Fig. 3), the training set is composed of all six doses of a single cell type. All six doses of the other two cell types can be used as validation data. The outcomes of model validation and selection are determined based on each of these twelve different subsets of validation data, separately. The schemes are named throughout the manuscript as Sln1, Sho1 and WT schemes depending on the cell type used for training.

**Fig. 4:**
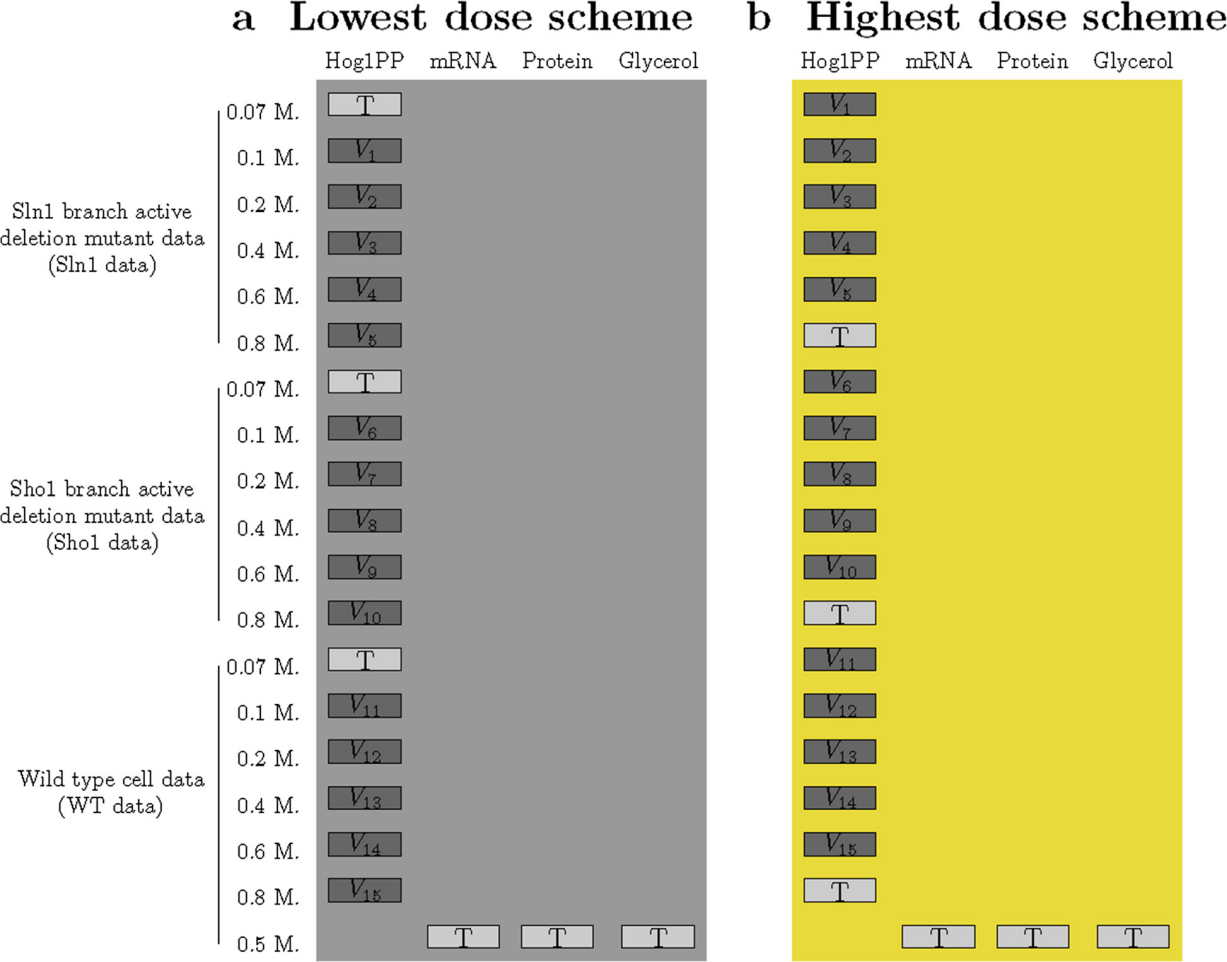
Scenario 2 partitioning schemes. Light gray colored boxes show parts of the data which we used as the training set (T) for parameter estimation. Dark gray colored boxes show parts which we used as validation sets (V). Different background colors represent different partitioning schemes and are consistent with the colors used in the graphs in the Results and discussion section. Each partitioning scheme offered the use of three subsets of the Hog1PP data as the training set. These are the lowest dose subset of each cell type in the lowest dose scheme and the highest of each in the highest dose scheme. The remaining fifteen subsets of the Hog1PP data could be used for validation, separately. These are the lowest dose subset of each cell type in (**a**) the lowest dose scheme and the highest of each in (**b**) the highest dose scheme

Our second scenario mimics the dose-response strategy. In this scenario, the training set is composed of one dose from each cell type. In the lowest dose scheme, only the data following a 0.07 M. NaCl shock are used for training (Fig. 4). In the highest dose scheme, only the data following a 0.8 M. NaCl shock are used for training. The remaining five doses from each cell type can be used as validation data. Similar to the first scenario, the outcomes of model validation and selection are determined based on each of these fifteen subsets of validation data, separately.

**Fig. 5:**
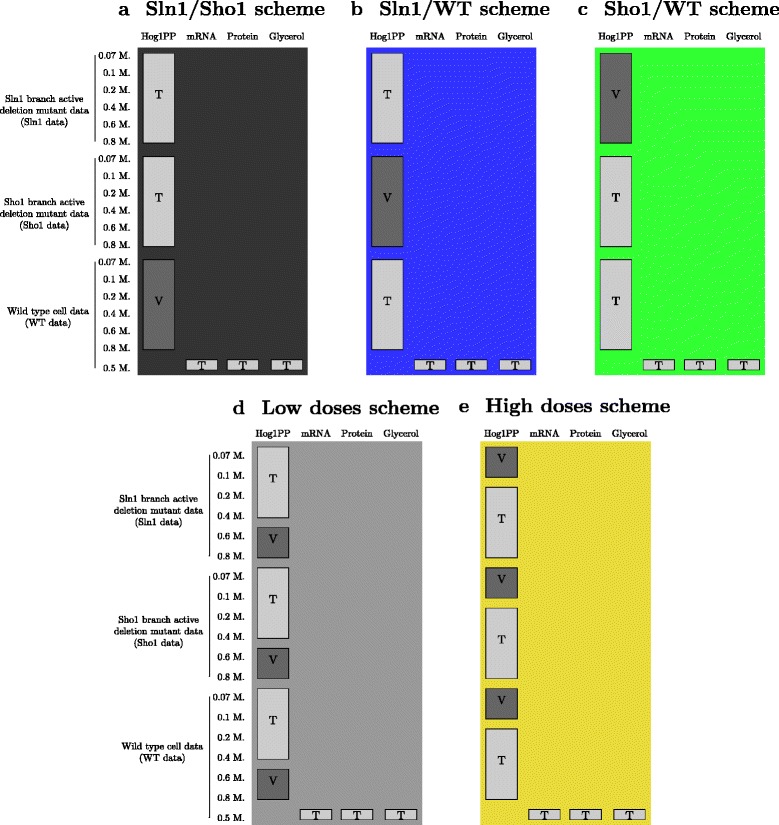
Partitioning schemes used in the adapted scenarios. Light gray colored boxes show parts of the data which we used as the training set (T) for parameter estimation. Dark gray colored boxes show parts which we used as the validation set (V). Different background colors represent different partitioning schemes and are consistent with the colors used in the graphs in the Results and discussion section. Each partitioning scheme offers the use of twelve subsets of the Hog1PP data as the training set and the remaining six subsets as the validation set. The training set included either (**a**) Sln1 & Sho1 data, (**b**) Sln1 & WT data, (**c**) Sho1 & WT data, (**d**) the lowest four doses from each cell type or (**e**) the highest four doses from each cell type

Lastly, we introduce variation in the training sets. We update our first scenario in such a way that in each partitioning scheme (Fig. 5a-c), we use data from two cell types for training. All six doses from the remaining cell type can be used as validation data. We make consensus decisions on model validation and selection considering all the validation subsets. The schemes are named throughout the manuscript as Sln1/Sho1, Sln1/WT and Sho1/WT schemes depending on the pair of training cell types. We update our second scenario in such a way that in each partitioning scheme (Fig. 5d-e), we use either data from the four highest or four lowest doses from each cell type for training. The remaining two doses from each cell type can be used as validation data. Similar to the first updated scenario we make consensus decisions using all validation subsets at once. The schemes are named as low doses and high doses schemes based on the doses used in the training set. This way, we can obtain five schemes (Fig. 5) each of which uses twelve subsets of the phosphorylated Hog1 (Hog1PP) data for training and the remaining six subsets for validation. Therefore, these five schemes can be compared to the stratified cross-validation scheme which also makes use of twelve subsets of Hog1PP data in each training set.

#### Stratified random cross-validation scheme

**Fig. 6:**
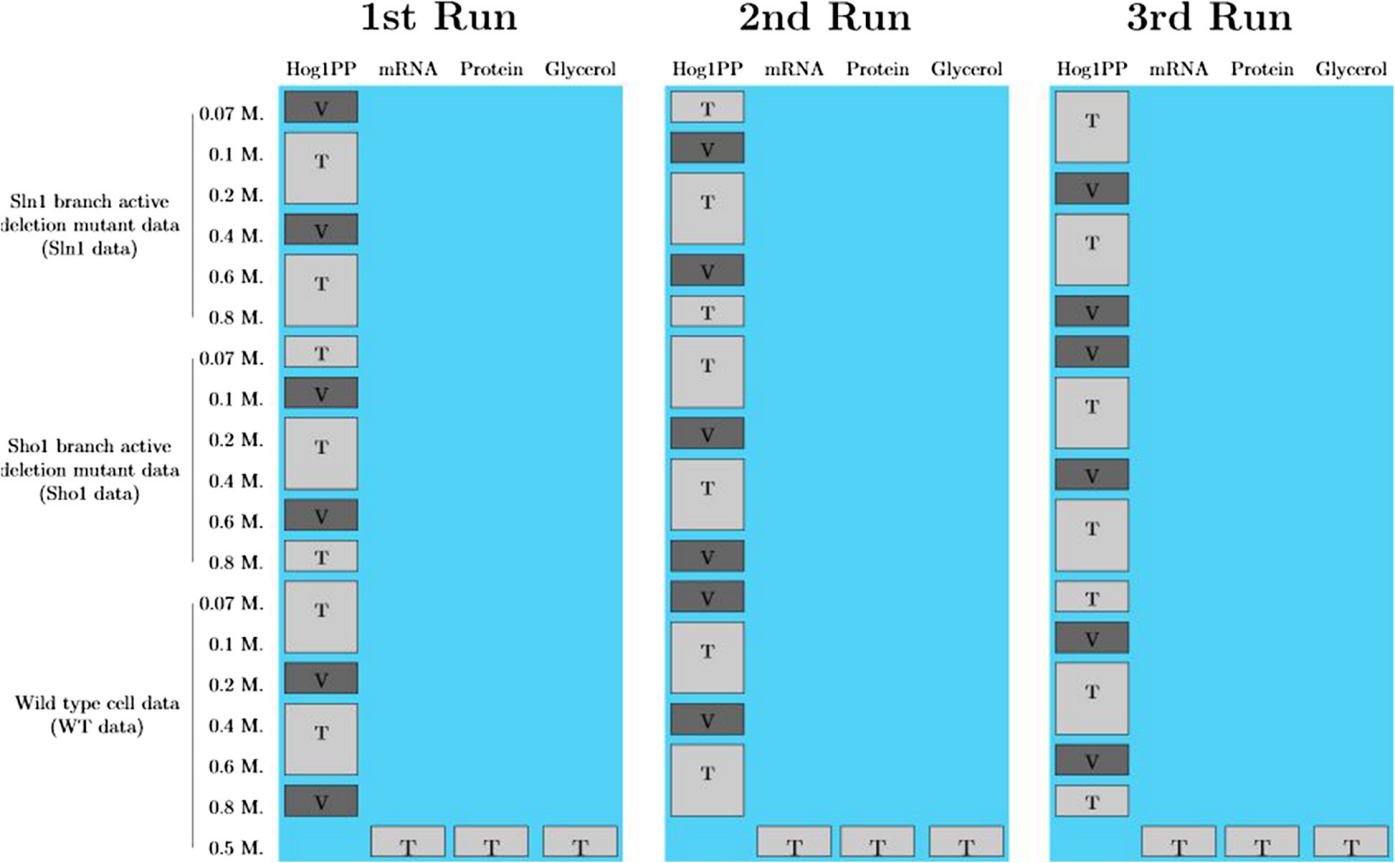
Stratified random cross-validation scheme (SRCV). Light gray colored boxes show parts of the data which we used as training sets (T) for parameter estimation. Dark gray colored boxes show parts which we used as validation sets (V). In each of the three runs, the training and the validation sets change as indicated in these graphs

In a random cross-validation scheme, there are no pre-defined partitions, unlike the hold-out partitioning schemes. Here, we implement stratified random cross-validation which is a specific type of cross-validation in which the training sets can be forced to follow a certain structure. We randomly partition the data into training and validation sets, in three different runs. In each run, we force the training sets to include the same amount of data from each cell type and dose level. We estimate the parameters and also calculate the measures we that we use for the analysis of the simulations (further explained in the next subsection), at each run. Later, we make consensus decisions using the average of these measures that were evaluated at each run. The different partitioning schemes applied in each run can be seen in Fig. 6.

Stratified cross-validation is based on the idea of providing training sets in which all classes of data are represented approximately equally. In our case, different classes of data are different cell types and different doses. This makes it a suitable scheme for our purposes, because we anticipate biased parameter estimates and hence, biased model validation and selection when training sets are dominated by certain classes of data. A stratified scheme would typically avoid such a problem and would give more robust results across different runs of cross-validation compared to other cross-validation approaches.

### Parameter estimation

For each partitioning scheme, we estimated the parameters of both the true model (the model that we accepted as the ground truth and generated the data upon that) and the simplified model (the model that lacked one of the important regulatory interactions in the true model which can be seen in Fig. 1). We repeated the parameter estimation process by using 100 different realizations of the data. The estimation of the parameters required the minimization of the difference between the data and model predictions. We carried out the minimization using the local minimizer ‘lsqnonlin’ function of Matlab [27, 28]. We considered a local optimizer to be sufficient since we work with generated data and could use the true values of parameters as starting points.

In the case of real data, true values of the parameters are not known. However, usually there is prior information on the ranges of the values that the parameters can take. In such a situation, uniformly distributed random starting points can be generated in these ranges and optimization with lower and upper bounds can be performed starting from the different initial points, aiming to find the same minimum in a sufficient number of runs. We took this approach for a single example noise realization. We assumed that the parameter ranges span intervals that are twice as big as the true values of the parameters. We started the optimization from 80 different starting points and could achieve the same minimum with the one achieved when the true values of the parameters were used as the starting point, in 20 % of the runs. The correlation between the parameter estimate vectors of these runs was above 0.99. This finding confirmed that performing parameter estimation in a more realistic setting does not affect the minimum achieved if there is good prior information on the parameter ranges. Therefore, we use fixed starting points (true values of the parameters) throughout the study due to its substantial advantage in computational power.

### Measures used for the analysis of the simulations

We analyzed five main features from the simulations, namely the amount of bias in the parameter estimates, the predictive power of the models on the validation datasets, the number of wrong decisions in which the simplified model structure was selected over the true model structure and the distance between the predicted profiles by the true and the simplified model structures (model separation).

We use normalized bias (*nBi*) as a measure of the bias in each estimated parameter (Equation 1). The median of its distribution across different noise realizations gives us the median amount of bias in each parameter estimated in a certain scheme.

(1) $$\begin{array}{*{20}l} & {\mathit{nBi}}^{i}_{j} = \frac{\left|{p^{i}_{j}} - p^{i}_{true}\right|}{p^{i}_{true}}\times 100 \\ & i = \text{1:20 index for parameters in the model} \\ & j = \text{1:100 index for noise realizations}  \end{array} $$

We use normalized standard deviation of parameter estimates as a measure of identifiability levels of parameters. We obtain the standard deviation of the estimates by calculating the Fisher Information Matrix.

We quantify the lack of good predictive power of models by using percentage errors. Percentage error is the percentage of the sum of squares of the prediction error to the sum of squares of validation data (Equation 2). Model selection between the two models gives wrong results when *PE*_*T*_>*PE*_*S*_, meaning that the simplified model gives lower prediction error than the true model structure.

(2) $$  \begin{aligned} & PE=\frac{{\sum_{i}^{I}}\left(\frac{\sum_{j}^{15}{\left(x_{ijk}-\hat x_{ijk}\right)^{2}}}{\sum_{j}^{15}{x_{ijk}^{2}}} \times 100\right)}{I}\\ & i = \text{1:I index for validation subsets of Hog1PP data}\\ & j = \text{1:15 index for time points}\\ & I = \text{total number of Hog1PP subsets used for validation} \end{aligned}   $$

**Fig. 7:**
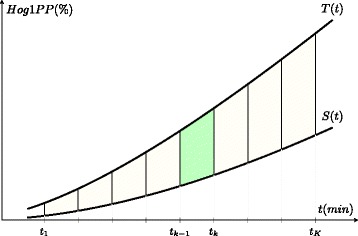
Trapezoidal Rule. The figure explains the trapezoidal rule visually. The green shaded area refers to the area of the (*k*−1)^*th*^ trapezoid. The total area of the trapezoids is equal to *Δ* *T* *S*_*i*_ in Equation 3

The difference between the true and the simplified model predictions (*ΔTS*) can be calculated by using the trapezoidal rule as in Equation 3. With this method, the area between two curves can be approximated as a series of trapezoids (see Fig. 7). The sum of the areas of the trapezoids provide a good approximation of the area between the curves when the number of trapezoids are sufficiently high. Here, the two curves are the profiles of the Hog1PP predicted by the true and the simplified model structures. We normalize the calculated area with respect to the maximum of the Hog1PP data in the corresponding validation subset. Large areas between the two curves mean that the separation of the two model structures is easier. Therefore, when correct model selection decisions are given, model separation (*ΔTS*) can be used as an additional criteria of enhanced model selection.

(3) $$  \begin{aligned} & \Delta {TS}_{i} =\frac{\sum_{k}^{K-1}{\frac{\left|T\left(t_{k+1}\right) - S\left(t_{k+1}\right)\right| + \left|T\left(t_{k}\right) - S\left(t_{k}\right)\right|}{2}.\left(t_{k+1}-t_{k}\right)}}{max\left(x_{ij}\right)}\\ & \Delta TS = \frac{{\sum_{i}^{I}}{\Delta {TS}_{i}}}{I}\\ & \text{T: numerical values of the Hog1PP predictions by the}\\ &\quad\text{true model structure}\\ & \text{S: numerical values of the Hog1PP predictions by the}\\ &\quad\text{simplified model structure}\\ & \text{k} = \text{1:K-1 index for trapezoids}\\ & \text{i} = \text{1:I index for validation subsets of Hog1PP data}\\ & \text{j} = \text{1:15 index for time points}\\ & \text{I} = \text{total number of validation subsets} \end{aligned}   $$

## Results and discussion

### Scenario 1: partitioning of data from different cell types

**Fig. 8:**
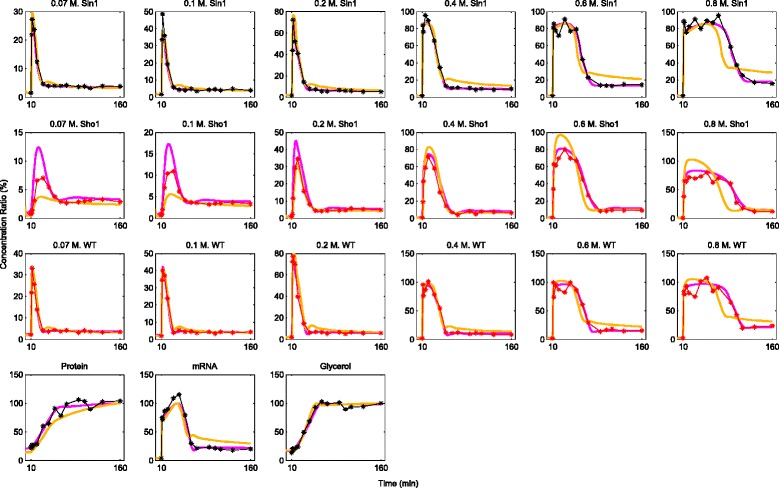
Fit and predictions obtained on a single realization of data in the Sln1 scheme. Black and red points (connected by lines of the same color) refer to data points which were used for parameter estimation and validation, respectively. In this example, all doses of Sln1 data and the data on the downstream species (protein, mRNA and internal glycerol) were used for parameter estimation. The magenta lines show the profiles obtained (both fit and prediction) by using the true model for the parameter estimation. The orange lines belong to the profiles obtained by the simplified model structure. All concentrations are given in percentages. The top three rows are for the Hog1PP data. The titles for each graph show the dose and the cell type related to the experiment in which the Hog1PP data was collected. The last row of graphs give the concentration ratios for the downstream species. The associated data was collected in a single experiment with WT cells following a 0.5 M. NaCl shock

Firstly, we would like to stress that in all of our simulations, we observed very good fit of the true model structure to the data. Additionally, our emphasis in this work is on model validation and selection using validation datasets which were excluded from the training set. Therefore, we do not present detailed analysis of the quality of model fits. Only in Fig. 8 and Additional file 1: Figure S2, we present the model fits together with the predictions in two examples. We should also mention that the term ’prediction’ always refers to predictions on validation datasets, throughout the text. Finally, we present our results on both percentage error (PE) and model separation (*Δ*TS) using a box plot representation. With this representation, each box plot shows the distribution of the associated measure across the 100 different noise realizations. For example, in the case of percentage error, the median of this distribution gives an idea on how high the prediction errors are in general. In addition, the box plots show also the outliers with relatively high prediction errors by the red points outside the boxes.

**Fig. 9:**
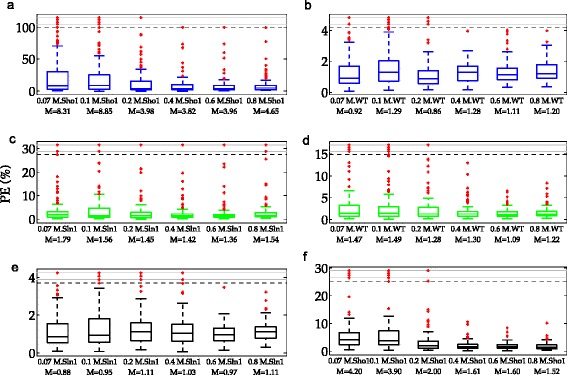
Percentage prediction errors (PE) of the true model structure in scenario 1. Each box plot shows the distribution of PE over 100 different realizations of the data. The red dots indicate the outliers which lie outside approximately 99.3 % coverage if the data is normally distributed. They indicate realizations with relatively higher PE. Blue, green and black boxes refer to Sln1, Sho1, and WT schemes. Each row in the figure corresponds to a single scheme. The labels on the x-axis show the specific dose and the cell type of the data on which the validation was performed. The labels indicate also the medians of the PE distribution summarized visually by the box plots. In each graph, the ten realizations with the highest PE are located above the black dashed line. The region above this line is compressed for visual ease. **a** PE obtained on Sho1 validation subsets in the Sln1 scheme. **b** PE obtained on WT validation subsets in the Sln1 scheme. **c** PE obtained on Sln1 validation subsets in the Sho1 scheme. **d** PE obtained on WT validation subsets in the Sho1 scheme. **e** PE obtained on Sln1 validation subsets in the WT scheme. **f** PE obtained on Sho1 validation subsets in the WT scheme

When only data from the Sln1 branch active deletion mutant (Sln1 data) is used for parameter estimation, validation using the Sho1 branch active deletion mutant data (Sho1 data) can be very misleading. This is because models trained by using Sln1 data results in bad predictions on the Sho1 data. On the other hand, the same models can achieve reasonable predictions on the WT data (See Fig. 8 for an example). This can be seen from the distribution of the percentage prediction errors represented by box plots for each validation set in Fig. 9a and b.

Existence of realizations with very high prediction errors in box plots with low medians shows that extremely bad predictions can occur even when the median prediction error is not very high. Examples of this can be observed also in the Sho1 scheme shown in Fig. 9c and d. Indeed, the models trained by using only the Sho1 data can lead to extremely high prediction errors both on Sln1 and WT data. This can be seen from the existence of realizations with a percentage prediction error above 30 % and 15 %, respectively. On the other hand, models trained by using only the WT data perform well in predicting the Sln1 data but not the Sho1 data (maximum of medians = 1.11 % vs 4.20 % in Fig. 9e and f). However, they are still better than those obtained by the models trained by the Sln1 data (maximum of medians = 4.20 % vs 8.85 % in Fig. 9f and a).

As a summary of the observations on the predictive power, we can say two things. Firstly, models trained by using only the data from one of the deletion mutants is poor in predicting the data from the other. Secondly, models trained by using the data from the WT cell can predict the data from one of the deletion mutants better than the other one. The poor predictions might easily lead to misleading decisions on model validation. True model structures might fail to be validated due to weak predictive power of some partitioning schemes. To study the reasons leading to weak predictive power we investigated the parameter estimation quality.

**Fig. 10:**
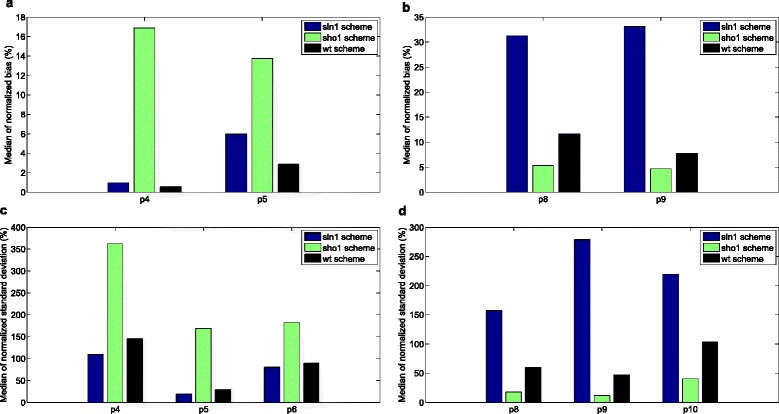
Normalized bias and standard deviation of branch parameters. Bar graphs show the median of the normalized bias and standard deviation of parameters across all noise realizations. Only some of the branch parameters are shown in the figure. Parameters p4-p5-p6 play a role in the Sln1 branch and parameters p8-p9-p10 are in the Sho1 branch. Blue, green and black refers to the Sln1, Sho1 and WT schemes respectively. **a** Median of normalized bias in Sln1 branch parameters. **b** Median of normalized bias in Sho1 branch parameters. **c** Median of normalized standard deviation in Sln1 branch parameters. **d** Median of normalized standard deviation in Sho1 branch parameters

We measured the parameter estimation quality by using the normalized bias of each parameter. The median of this measure across all noise realizations shows how well the parameter was estimated in general in a certain scheme. In Fig. 10b, we see that the parameters related to the complex formation of Sho1 and Pbs2 proteins and this complex’ phosphorylation, p8 and p9, were predicted with very high bias in the Sln1 scheme. (see the yellow region in Fig. 1). This means that when the Sln1 data is used for model training, we estimate the Sho1 branch parameters with a very high uncertainty with a median bias of 31 % and 33 %, respectively. The same reasoning is valid also for the estimation of two of the parameters related to the phosphorylation of the Pbs2 protein, p4 and p5. The median bias for these parameters (see the gray region in Fig. 1) were found to be 17 % and 14 %, respectively (see Fig. 10a). There is an interesting difference between the estimation quality of the parameters in the two different branches, though. We could decrease the bias of the Sln1 branch parameters considerably when we used the WT data for training the model. However, the level of bias in the Sho1 branch parameters was still relatively high in the WT scheme compared to the Sho1 scheme. Similarly, the identifiability analysis (see Fig. 10c) shows that training the model on the WT data results in similar standard deviations in the Sln1 branch parameters when compared to the standard deviations obtained in the Sln1 scheme. However, the standard deviations of the Sho1 branch parameters are much lower in the Sho1 scheme compared to those obtained in the WT scheme (see Fig. 10d) which suggests improved identifiability in the Sho1 scheme. Therefore, the Sln1 data could be predicted well in the WT scheme whereas the prediction of the Sho1 data was still problematic. As a further investigation on the system dynamics, we tuned one of the branch parameters each time within a range limited by the minimum and maximum of their estimated values. This allowed us to confirm the deteriorating effect of biased branch parameters on the predictions (data not shown).

The asymmetrical behaviour of the predictive power (that is, the WT and the Sln1 schemes were good in predicting Sln1 and WT validation data, respectively but none of them could achieve good predictions on the Sho1 validation dataset) stems from an underlying biological property which is the inequality of the two phosphorylation branches in the model. Although the two branches (Fig. 1) act redundantly for the ultimate goal of Hog1 protein phosphorylation, the fluxes in each branch are not equal. As also mentioned in [25], the Sho1 branch active deletion mutant produces less output in terms of phosphorylated Hog1 protein. This biological fact manifests itself also in the data. The WT data is characterized more by the activity in the Sln1 activation branch rather than the Sho1 branch. In other words, the Hog1PP levels in the WT cell are affected more by the changes in the Sln1 branch parameters than by the changes in the Sho1 branch parameters. Therefore, the WT data can substitute for the Sln1 data for training the models. However, the cost of excluding the Sho1 data from the training set is higher due to the asymmetry we mentioned above. The Sho1 branch parameters are weakly identifiable when the Sho1 data is not used for parameter estimation. This asymmetry in the information content of the data is clearly the output of the pathway machinery. This machinery is summarized into a model with a model structure and parameter values. Therefore, the decisions of model validation using a hold-out strategy is dependent on the underlying biological properties (the asymmetrical branch structure in this particular example) and reflections of these properties in the model parameters (parameter values that allow less flux in one of the branches). The data partitioning task, hence, proves to be a difficult one since the prior knowledge about the underlying biology would never be complete.

Another important observation is the NaCl dose dependency of the predictive power using the Sho1 data. The predictive power using Sho1 data was especially lower in the lowest two doses compared to the higher doses as can be seen in Fig. 9a and f (maximum of the medians 8.85 % vs. 4.65 % in the Sln1 scheme and 4.20 % vs. 1.52 % in the WT scheme).

**Fig. 11:**
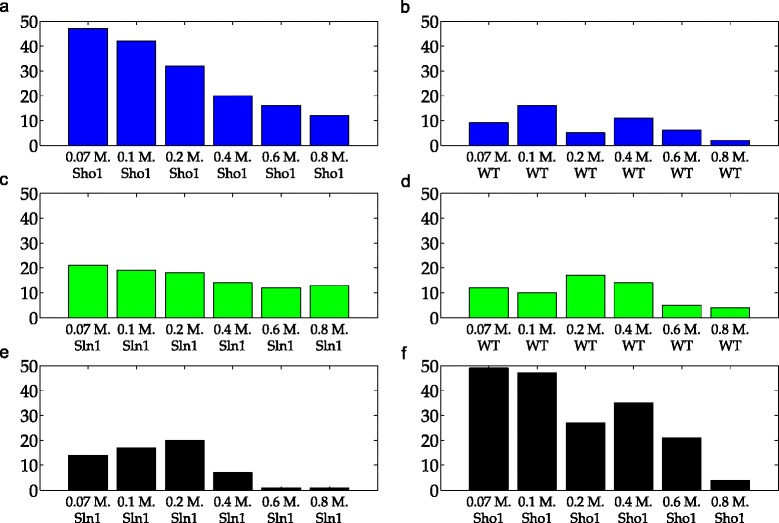
Number of wrong decisions in scenario 1. Bars show the number of realizations in which the simplified model gave lower residuals than the true model structure and therefore, was wrongly selected over the true model structure. Blue, green and black bars refer to Sln1, Sho1, and WT schemes. Each row in the figure corresponds to a single scheme. The labels on the x-axis show the specific dose and the cell type of the data on which the validation was performed. **a** Number of wrong decisions using Sho1 validation subsets in the Sln1 scheme. **b** Number of wrong decisions using WT validation subsets in the Sln1 scheme. **c** Number of wrong decisions using Sln1 validation subsets in the Sho1 scheme. **d** Number of wrong decisions using WT validation subsets in the Sho1 scheme. **e** Number of wrong decisions using Sln1 validation subsets in the WT scheme. **f** Number of wrong decisions using Sho1 validation subsets in the WT scheme

The asymmetry in the contribution of the Sln1 and the Sho1 branches to the phosphorylation of the Hog1 protein also has consequences for model selection. Figure 11 shows the number of wrong decisions given on each validation subset in each of these three partitioning schemes. We see that in a high number of realizations, the simplified model structure was selected over the true model structure when the Sho1 data was used for validation (Fig. 11a and f). On the other hand, using only the Sho1 data for training also resulted in an increased number of wrong decisions on the Sln1 data compared to the WT scheme (minimum number of wrong decisions 12 vs. 1 in Fig. 11c and e).

In this section, we focused on partitioning schemes which use the data from only one cell type for parameter estimation. Our results show the importance of having a variety of different validation sets. This is because decisions of model validation and selection vary considerably depending on the experimental conditions of different validation sets due to unknown values of the underlying parameters.

### Scenario 2: partitioning of data in different doses

**Fig. 12:**
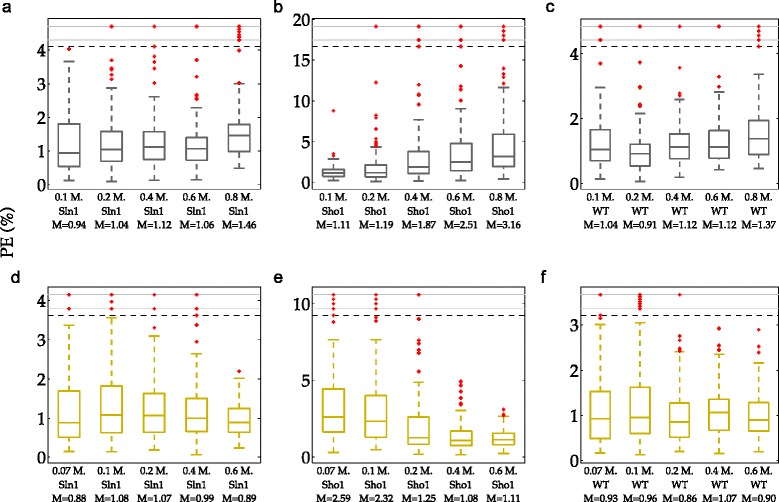
Percentage prediction errors (PE) of the true model structure in scenario 2. Each box plot shows the distribution of PE over 100 different realizations of the data. The red dots indicate the outliers which lie outside approximately 99.3 % coverage if the data is normally distributed. Gray and yellow boxes refer to the lowest and the highest dose schemes, respectively. Each row in the figure corresponds to a single scheme. The labels on the x-axis show the specific dose and the cell type of the data on which the validation was performed. The labels indicate also the medians of the PE distribution summarized visually by the box plots. In each graph, the ten realizations with the highest PE are located above the black dashed line. The region above this line is compressed for visual ease. **a** PE obtained on Sln1 validation subsets in the lowest dose scheme. **b** PE obtained on Sho1 validation subsets in the lowest dose scheme. **c** PE obtained on WT validation subsets in the lowest dose scheme. **d** PE obtained on Sln1 validation subsets in the highest dose scheme. **e** PE obtained on Sho1 validation subsets in the highest dose scheme. **f** PE obtained on WT validation subsets in the highest dose scheme

In the second scenario, where we use different doses as training sets, we see a change of predictive power on Sho1 validation data (see Fig. 12b and e). When the lowest dose data from all three cell types are used for training, the predictive power on the Sho1 data decreases with increasing dose (median 1.11 % vs. 3.16 % on 0.1 M. and 0.8 M. dose levels, shown in Fig. 12b). Also, when the highest dose scheme is used, the predictive power increases with increasing dose (median 2.59 % vs. 1.11 % on 0.07 M. and 0.6 M. dose levels, shown in Fig. 12e).

These results showed us that predictive power becomes lower with increasing distances between the training and the validation sets, where the distance is measured in terms of the dose of the triggering chemical. This means that the risk of invalidating the true model structure increases when the validation set is too distant from the training set. However, the limits between which the model parameters stay applicable depend very much also on the cell type as we have observed. The predictive power on the Sho1 data deteriorated more rapidly compared to the other cell types. These observations helped us to identify a serious pitfall of dose-response strategy: as long as we do not have realistic prior information on the limits for which we expect the estimated values of the model parameters to be applicable, we face the risk of invalidating a true model structure by over-challenging the model. Unfortunately, determination of the limits is not possible beforehand since it depends on the underlying biological properties which will never be completely known.

**Fig. 13:**
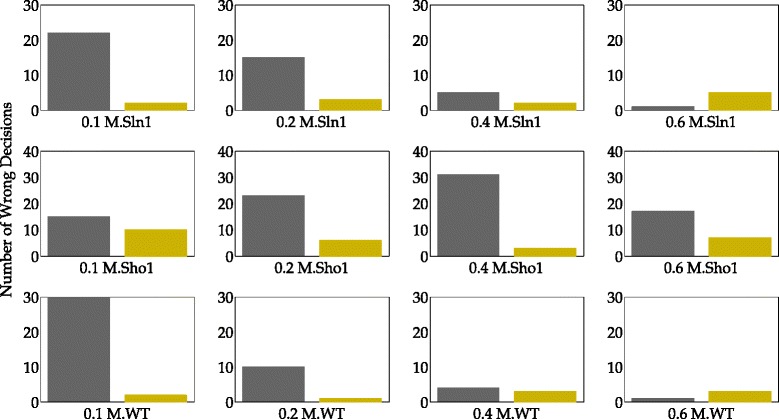
Number of wrong decisions in scenario 2. Bars show the number of realizations in which the simplified model gave lower residuals than the true model structure and therefore, was wrongly selected over the true model structure. Gray and yellow bars refer to the lowest and the highest dose schemes. The labels on the x-axis show the specific dose and the cell type of the data on which the validation was performed. Here, only the twelve validation subsets which could be used in both the lowest and the highest schemes are shown

**Fig. 14:**
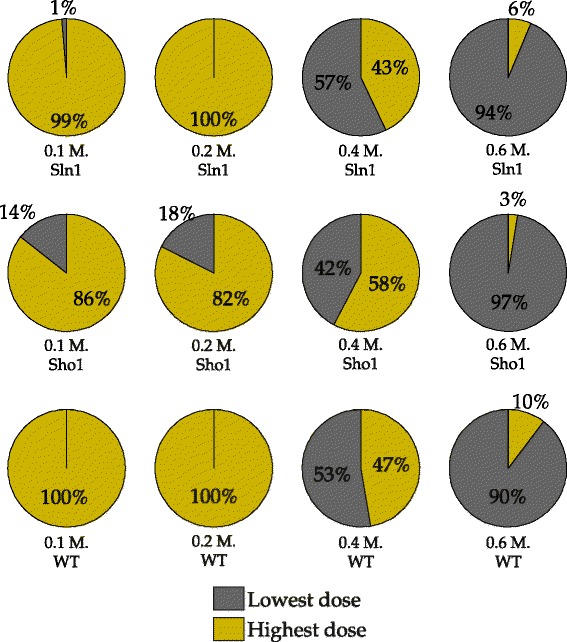
Comparison of model separation by the two schemes. Each pie chart shows the percentage of correct decisions, where the model separation achieved by a certain scheme is better than the other scheme. The labels on the x-axis show the specific dose and the cell type of the data on which the validation was performed. Gray and yellow colors in the charts refer to the lowest and the highest dose schemes. Here, only the twelve validation subsets which could be used in both the lowest and the highest schemes are shown. For example, when the 0.1 M. Sln1 data was used for validation, in 99 % of the realizations in which a correct decision was given by both schemes, higher distance between the predictions of the true and the simplified model structures was achieved in the highest dose scheme than the lowest dose scheme

When it comes to model selection, we face a different challenge. Figure 13 shows the number of realizations in which the simplified model structure was selected over the true model structure. For example, the lowest dose scheme results in 22 wrong decisions whereas the highest dose scheme results in only 2 wrong decisions when the 0.1 M. Sln1 dataset is used as validation dataset as can be seen in the upper left hand side corner of Fig. 13. Here, only the results on validation sets that can be used in both schemes are shown because our focus is on comparing the performance of two different schemes on shared validation sets. The most important observation from the figure is that the number of wrong decisions by the lowest scheme is higher on the 0.1 M. - 0.2 M. Sln1 and WT data compared to the highest dose scheme. The number of wrong decisions by the lowest scheme is very high (22 and 30 on the Sln1 and WT validation data, respectively) especially on the 0.1 M. dose which is very close to the 0.07 M. dose where the models were trained. In addition, we see that the highest scheme gives a slightly higher number of wrong decisions compared to the lowest dose scheme on the 0.6 M. Sln1 and WT data. These observations suggest that model selection is problematic when the training and validation sets are too close to each other. We looked further at the model separation between the true and the simplified models (see Fig. 14) to investigate the separation between the two model structures in higher resolution.

In cases where the differences between the number of wrong decisions is too low for a meaningful comparison, the model separation, *Δ*TS is more informative. Figure 14 shows the percentage of the realizations in which one specific scheme resulted in better model separation than the other scheme. The percentages are based on the number of realizations in which a correct decision was made by using both the lowest and the highest dose schemes. For example, we know that both schemes result in a correct decision in 77 realizations of the Sln1 data at 0.1 M. NaCl shock (data not shown). The first pie chart in Fig. 14 shows that in 99 % of these 77 realizations, the highest dose scheme resulted in better separation between the two model structures than the lowest dose scheme. As can be seen from this figure, model separation obtained by the highest dose scheme is higher than that obtained by the lowest dose scheme in almost all realizations of 0.1 M. - 0.2 M. Sln1 and WT data. At 0.6 M. dose, the situation is reverse and the lowest scheme provides a better separation of the two model structures, in most of the realizations of all three cell types. These findings support the observation we made from the number of wrong decisions: model selection becomes problematic with too close training and validation sets. This is mainly because the simplified model might also predict well in the close proximity of the training dose (See Additional file 1: Figure S1). However, it will perform worse than the true model structure as the training and validation sets become more distant from each other. However, too much distance can also pose a problem for model selection due to increased uncertainty in the predictive power. Uncertainty in the predictions shows that different noise realizations can either give very good or very bad predictions. High levels of uncertainty reveals itself in the wide box plots of especially Sho1 validation data in Fig. 12b, 12e and Additional file 1: Figure S1, showing a wide dispersion of predictive power across different noise realizations. In these regions where the uncertainty is high, it becomes more difficult to anticipate the predictive powers of the true and the simplified model structures on a single noise realization. This hampers also model selection. Using Sho1 validation data results in such a situation where uncertainty is very high at certain doses. This is why the trends in model selection that we have presented in this section cannot be observed on the Sho1 validation data as sharply as on the other cell types.

We can understand the risks associated with high uncertainty in a hold-out strategy, if we remember that in a single real experiment we have only one realization of noise. The outcomes of both model validation and selection depend highly on the specific noise realization in the data but we have only one realization available. This means that it is highly probable that we end up in wrong decisions just due to experimental noise. Therefore, we need partitioning schemes that minimize the effect of idiosyncratic noise realisations and lead to similar decisions for all of them. The stratified random cross validation scheme is promising in this sense as we will explain in the following section.

### Introducing variation in the training and the test data

In the previous sections, we showed the pitfalls that we might come across if we use single doses or single cell types as validation data. Therefore, we stress the importance of consensus results obtained from a collection of different validation sets. In this section, we take it one step further and introduce variation of experimental conditions also in the training data. We do it in three different ways as described by the adapted scenarios and the stratified cross validation scheme in the Methods section. First, we include two different cell types in the training data, namely in the Sln1/Sho1, Sln1/WT and Sho1/WT schemes. Second, we include four different doses from each cell type in the training data, namely in the low doses and the high doses schemes. These are examples of hold-out validation strategies just like the previous two scenarios. However, unlike those, the training and the validation sets include a variety of different cell types or doses. The third way is not an example of a hold-out strategy. It is the stratified random cross-validation (SRCV) scheme about which we have given the details in the Methods section. With this approach we can introduce variation in the training and validation sets in terms of both cell types and doses at the same time.

**Fig. 15:**
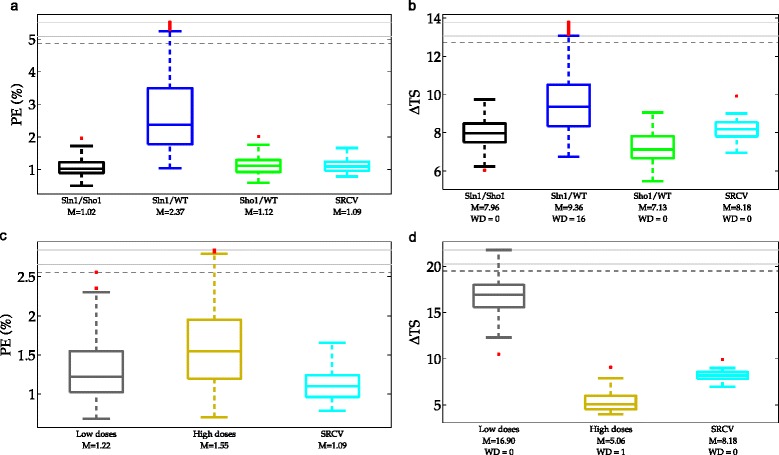
Percentage prediction errors (PE) and model separation (*Δ*TS) in the adapted scenarios. Each box plot shows the distribution of PE or *Δ*TS over 100 different realizations of the data obtained in a single scheme. The red dots indicate the outliers which lie outside approximately 99.3 % coverage if the data is normally distributed. Black, blue and green boxes in the first row of graphs refer to the Sln1/Sho1, Sln1/WT and Sho1/WT schemes. Cyan boxes refer to the stratified cross-validation (SRCV) schemes. Gray and yellow boxes in the second row refer to the low doses and high doses schemes. The labels on the x-axis indicate the medians of the PE or *Δ*TS distribution summarized visually by the box plots. The axis labels in the *Δ*TS graphs show also the number of wrong decisions given in each scheme. In each graph, ten realizations with the highest PE or *Δ*TS are located above the black dashed line. The region above this line is compressed for visual ease. **a** PE obtained in adapted cell type scenario. **b** *Δ*TS obtained in adapted cell type scenario. **c** PE obtained in adapted dose scenario. **d** *Δ*TS obtained in adapted dose scenario

Firstly, we compare the schemes in which the training set includes different cell types. The most important observation regarding these three schemes is the low predictive power in the Sln1/WT scheme as can be seen in Fig. 15a. This shows that when the models are trained without using the Sho1 data, validating them on Sho1 data is risky. On the contrary, when the Sln1 data is missing in the training set, we do not observe such low predictive power. The reasons for this can be traced back to the asymmetrical branch structure that we explained in detail in the Scenario 1 section. Therefore, we do not discuss those here again.

In addition to the risks associated with model validation, the Sln1/WT scheme performes poorly also in model selection with 16 wrong decisions. Therefore, we conclude that the Sln1/WT scheme is not a good scheme for model validation and selection whereas the Sln1/Sho1 and the Sho1/WT are sensible partitioning schemes. The SRCV scheme results in prediction errors that are comparable with the sensible partitioning schemes (Fig. 15a). Furthermore, it results in no wrong decisions and it gives the highest model separation compared to the Sln1/Sho1 and Sho1/WT schemes which also result in all correct decisions (Fig. 15b). In addition to this, the predictive power of such a scheme is less dependent on the noise realization compared to the other schemes as can be seen from the smaller box plots in Fig. 15a. This indicates the low amount of uncertainty in the predictions.

When only doses were allowed to vary in the training set as in the case of the low doses and the high doses scheme, there was no significant difference in the predictive powers of the two schemes (Fig. 15c). This revealed that none of the schemes posed more risk of invalidating the true model structure compared to the other scheme. However, there was a large difference in the model separation achieved by the two schemes (median = 16.9 vs. 5.06 in the low and high doses schemes respectively, shown in Fig. 15d). This shows that the high doses scheme is unsuitable for model selection. A simulation showing weak model separation according to the highest dose scheme can be seen in Additional file 1: Figure S2. The SRCV scheme performed better than the unsuitable hold-out partitioning scheme for model selection (median *Δ*TS = 8.18) and the predictive power was in the range of the two hold-out partitioning schemes (Fig. 15c).

The observations explained above can also be anticipated from the identifiability levels. The standard deviations of parameters at all three runs of the SRCV scheme were comparable to those obtained in the sensible hold-out partitioning schemes for model validation (see Additional file 1: Figure S3) and were never higher than those obtained in the unsuitable schemes.

These results indicated that a stratified CV scheme is favorable for both model validation and selection. In most of the comparisons, it achieves predictive power and model separation as high as the optimal hold-out partitioning scheme. In addition, it leads to lower uncertainty which means that the outcomes of model validation and selection depend less on the specific noise realization. More importantly, it never performs worse than unsuitable hold-out partitioning schemes. The importance of this last statement lies in the fact that finding a sensible hold-out partitioning scheme can never be guaranteed. It depends highly on the biology and therefore, on the model structure and the model parameters most of which are typically unknown prior to modeling. Therefore, there are no rules that can be set beforehand to make the finding of sensible partitioning schemes certain. Those factors might hinder us from opting for a sensible scheme. However, SRCV offers a judicious and reliable partitioning scheme for which no biological knowledge is required. Its good performance relies on two properties. Firstly, it is iterative which means that it allows each piece of data to contribute as both training and validation datasets in an iterative manner and summarizes the results as the average of different iterations. Secondly it offers random stratified partitioning, so it allows fair partitioning of the data while it prevents from certain cell types or doses dominating the training data. Therefore, issues like parameter estimation and model validation/selection are not biased in a certain direction as an artifact of an underlying biological property of the system, in contrast to the hold-out validation schemes we have extensively investigated with this study. In addition, we achieve this by using a CV scheme with 3 folds and no repeats and hence, the computational time increases only three times compared to the hold-out schemes.

On the other hand, our additional simulations with two more complex models revealed that a prerequisite for model selection based on predictive power has to be mentioned. The first more complex model included one additional parameter (Hog1 dependent Fps1 degradation) whereas the second model included three additional parameters (Hog1 dependent Fps1 degradation, Fps1 production and protein dependent Fps1 degradation). We have found out that the additional parameters were estimated very close to 0. Median of the parameters changed between 0.8×10^−5^ and 0.4×10^−7^ in all of the schemes. This means that both of the complex models boiled down to the true model structure. Therefore, the differences in the prediction errors obtained with the complex and the true model structures were very small. For example, the difference in the prediction errors of the true and the complex model structure was, in average 2.03 % of the prediction error of the true model structure obtained with the Sln1/Sho1 scheme. However, this value was 146 % in our simulations with the simplified model structure. At this level of extreme similarity between the model structures, model selection based on tiny differences between the predictive powers of the models leads to random conclusions that are heavily dependent on the specific noise realization in the data. Instead in such situations, investigating the estimated values of the additional parameters gives clue if a more complex model is needed or not. From this we derive the following important conclusion regarding the scalability of our approach. The guidelines we present in our manuscript are aimed for more reliable decision making in model selection when the selection is made based on the predictive powers of the models. In cases where such model selection is not applicable, our guidelines are obviously not applicable either.

## Conclusions

Our results showed that the final decisions on model validation and selection can differ significantly when different hold-out partitioning schemes are employed. The selection of a sensible hold-out partitioning scheme that will help us to make reliable decisions depends on the biology. A good biological knowledge on the system and, hence, prior information on the structure and the true parameter values of the model are essential. Unfortunately, this is not possible in many instances. This turns the problem of finding a sensible partitioning scheme for model validation and selection into a Catch 22 problem. When the determination of a sensible partitioning scheme fails, we face the risk of invalidating true model structures or of failing to select the true model structure over the other alternatives. Examples of the first situation are very difficult to find in the literature, though, because, only successful validation examples are usually presented, leading to a ‘verification bias’. Furthermore, partitioning schemes that are sensible for model selection are not necessarily suitable for model validation. Datasets from very similar experimental conditions have only weak model selection capability whereas datasets from very diverse experimental conditions are not appropriate for model selection either due to high uncertainty in the predictions. However, using a proper cross-validation approach such as stratified random cross-validation can help us to overcome these problems while being independent of any prior biological knowledge.

With the SRCV approach, we can partition the data randomly into training and validation sets iteratively and arrive at consensus decisions by averaging over all different validation datasets. SRCV performs at least as well as sensible hold-out partitioning schemes for both model validation and selection. On top of that, this comes without the risk of opting for an incorrect partitioning scheme which would lead us to biased conclusions. Furthermore, the decisions given within a SRCV scheme are less affected by the specific realization of the experimental noise. Due to all these reasons that we mention, SRCV proves to be a judicious, unbiased and promising alternative to the hold-out validation strategy for the validation and selection of ODE based models.

## Additional file

Additional file 1
**Supporting figures.**
