# Supplementary material for: Validation and selection of ODE based systems biology models: how to arrive at more reliable decisions
Source: BMC Syst Biol. 2015 Jul 8;9:32. doi: 10.1186/s12918-015-0180-0 (PMC4493957; doi:10.1186/s12918-015-0180-0)
Supplement: Additional file 1 — Supporting figures. [file 12918_2015_180_MOESM1_ESM.pdf]

Supporting Figures

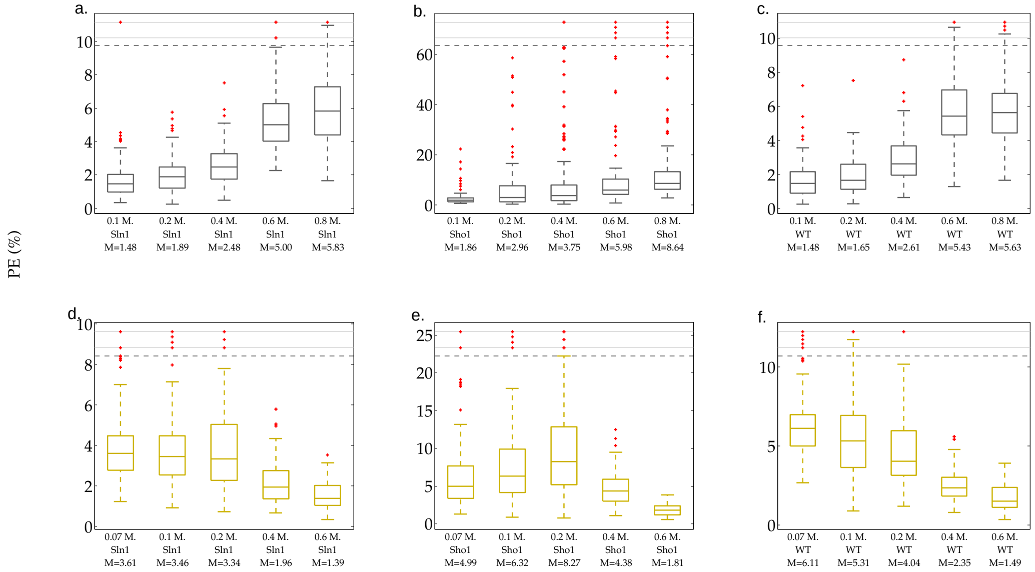

Figure S1: **Percentage prediction errors (PE) of the simplified model structure in scenario 2.** Each box plot shows the distribution of PE obtained with the simplified model structure over 100 different realizations of the data. The red dots show the outliers which lie outside approximately 99.3% coverage if the data is normally distributed. They indicate realizations with relatively higher PE. Gray and yellow boxes refer to the lowest and the highest dose schemes. Each row in the figure corresponds to a single scheme. The labels on the x-axis show the specific dose and the cell type of the data on which the validation was performed. The labels indicate also the medians of the PE distribution summarized visually by the box plots. In each graph, the ten realizations with the highest PE are located above the black dashed line. The region above this line is compressed for visual ease. **a.** PE obtained on Sln1 validation subsets in the lowest dose scheme. **b.** PE obtained on Sho1 validation subsets in the lowest dose scheme. **c.** PE obtained on WT validation subsets in the lowest dose scheme. **d.** PE obtained on Sln1 validation subsets in the highest dose scheme. **e.** PE obtained on Sho1 validation subsets in the highest dose scheme. **f.** PE obtained on WT validation subsets in the highest dose scheme.

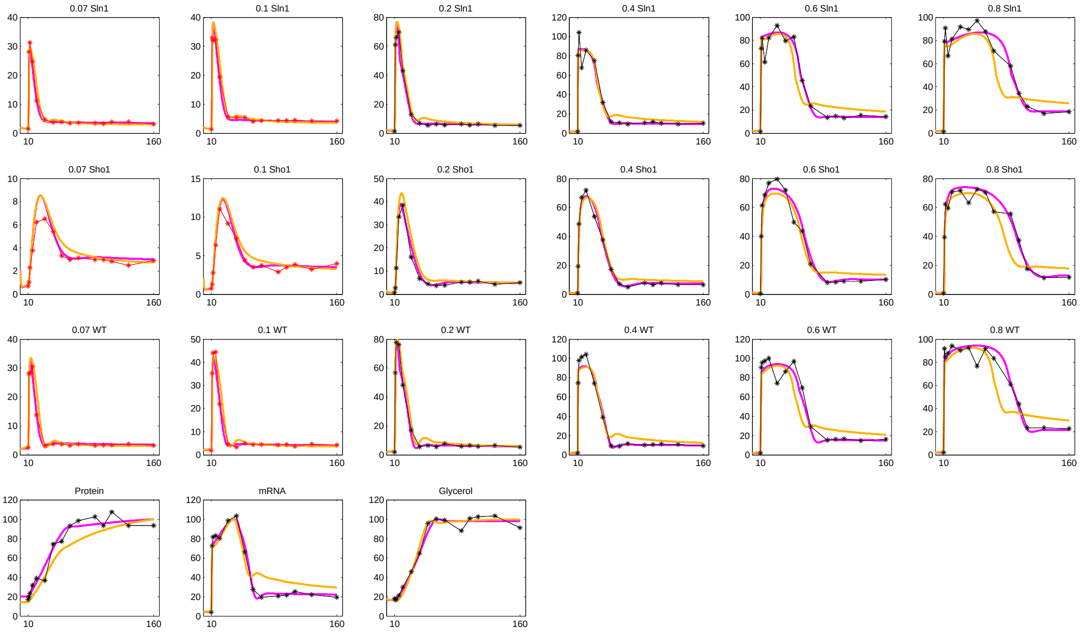

Figure S2: **Fit and predictions obtained on a single realization of data in the high doses scheme.** Black and red points refer to data points which were used for parameter estimation and validation, respectively. In this example simulation, data from doses between 0.2 M.-0.8 M. in each cell type and the data on the downstream species (protein, mRNA and internal glycerol) were used for parameter estimation. The magenta lines show the profiles obtained (both fit and prediction) by using the true model for the parameter estimation. The orange lines belong to the profiles obtained by the simplified model structure. All concentrations are given in percentages. Top three rows are for the Hog1PP data. The titles for each graph show the dose and the cell type related to the experiment in which the Hog1PP data was collected. On the other hand, the last row of graphs are for the downstream species and the associated data was collected in a single experiment with WT cell following 0.5 M. NaCl shock.

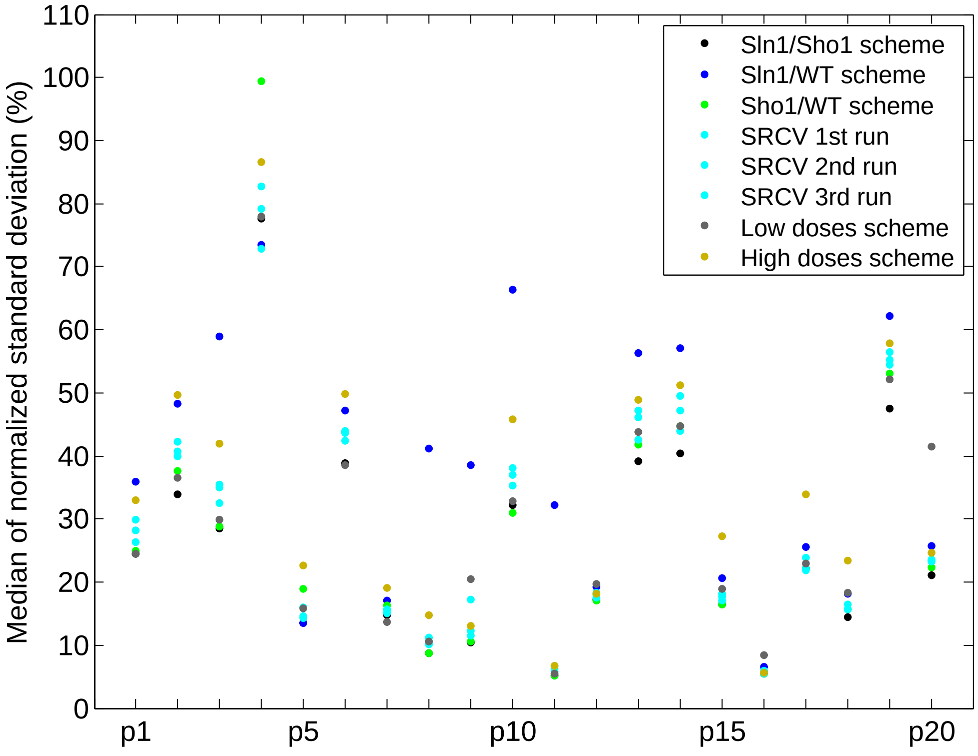

Figure S3: **Normalized standard deviation of parameters.** Each point shows the median of normalized standard deviation of each parameter obtained with the true model structure over 100 different realizations of the data. Different colors correspond to partitioning schemes in which the size of the training dataset is the same.
